# Supernatural-Functiolistic-Expialidocious: A Comparative Study Between Functional Neurological Symptom Disorder/Conversion Disorder and Other Psychiatric Conditions

**DOI:** 10.7759/cureus.100588

**Published:** 2026-01-01

**Authors:** Muhammad Noman K Wazir, Hoor S Kakakhel, Shuja S Majeed, Haseeba Mukhtar, Arslan R Ullah, Munsif Ali, Fakhria Wahid

**Affiliations:** 1 Psychiatry, Northwest General Hospital and Research Centre, Peshawar, PAK; 2 Medicine and Surgery, Northwest School of Medicine, Peshawar, PAK; 3 Rheumatology, Northwest General Hospital and Research Centre, Peshawar, PAK; 4 Community Medicine, Northwest School of Medicine, Peshawar, PAK; 5 Internal Medicine, Northwest General Hospital and Research Centre, Peshawar, PAK; 6 Internal Medicne, Northwest School of Medicine, Peshawar, PAK; 7 Occupational Health, Northwest General Hospital and Research Centre, Peshawar, PAK

**Keywords:** cross-cultural psychology, cultural mental health, dissociative (conversion) disorder, functional neurological symptom disorder, health system in pakistan, pakistan, psychiatry & mental health, psychological illnesses, psychology, southeast asia

## Abstract

Background

Functional neurological symptom disorder (FNSD) is a condition that affects the nervous system, disrupting how the brain and body communicate, leading to difficulties in movement and sensory perception. Our objective was to identify the stressors leading to a conversion disorder diagnosis, the frequency of medical and non-medical contacts, the type and average number of symptoms reported, and the local belief system surrounding the illness.

Methods

We collected data from 300 patients at the outpatient psychiatry clinic of North West General Hospital & Research Center, a tertiary care hospital in Peshawar, which serves both local residents and Afghan immigrants. The ethics committee of North West General Hospital and Research Centre approved all procedures involving patients or human subjects. Demographics and the required details were collected using predetermined tables. Group A included patients with a diagnosis of conversion disorder and group B included all other psychiatric illnesses. Comparative analysis was done using SPSS (IBM Inc., Armonk, New York) to analyze all collected data.

Results

Of the overall patients in group A, 69.33% believed in a spiritual cause, with or without a physical/psychiatric cause, for their complaints. 30.6% believed their complaints to be wholly physical or psychiatric in nature, with a female:male ratio (F:M) of 22.5:63.3%. A combination of spiritual and physical/psychiatric causes for the presenting complaints, were believed by 42.6% of the group, with F:M of 47.5:23.3%. The highest number of individual symptoms on average were reported in the group with marital problems at 6.7, followed by other form of stressors. Females, those residing in rural areas and were uneducated showed a greater number of symptoms. Females presented with overall more symptoms on average with a F:M of 2.5:2.2. Males who resided in rural areas presented with more symptoms on average, compared to those living in urban settings.

Conclusions

Our research showed that FNSD was more prevalent in young people, females, the uneducated and the ones living in rural areas. Females presented with more physical symptoms compared to males. Comparison with a control group of 150 other psychiatric patients, showed similar physical symptoms, but lesser in number compared to the FNSD group, they however had other similarities such as increase incidence in females, the uneducated and the ones from rural areas. The control group however showed more previous contact with physicians and psychiatrists compared to the FNSD group.

## Introduction

Functional neurological symptom disorder (FNSD), also known as conversion disorder (CD), is a condition that affects the nervous system's functioning. In this disorder, the brain and nerves fail to communicate effectively, disrupting the transmission of signals [1]. This "communication breakdown" can lead to physical symptoms such as difficulty moving limbs or problems with one or more senses and his/her ability to function [2]. Historically, CD was believed to be purely psychological, with emotional issues manifesting as physical symptoms [3]. However, it is now recognized as a distinct medical condition [2]. While psychological factors such as trauma, personal conflicts, and stress are commonly found in patients with conversion disorder, they do not appear in every case [4].

Conversion symptoms typically appear suddenly following a period of emotional or physical stress, or psychological conflict [5] and are believed to stem from the body's response to a stressful event - whether physical or emotional [6,7]. Some studies have suggested that neurological changes may be linked to the symptoms of this disorder [8]. Diagnosing conversion disorder (CD) involves recognizing specific signs common to the condition, along with conducting tests to exclude other possible causes of the symptoms [6].

Treatment often includes psychotherapy, hypnosis, and stress management techniques to address symptoms, along with treatment for any underlying psychological conditions [9]. Physical or occupational therapy may also be needed for the affected body part until the symptoms subside [10].

The symptoms of conversion disorder span both psychiatric and neurological domains. However, it's crucial to understand that these symptoms are genuine, not fabricated, and the patients are not consciously pretending [2]. Studies suggest that first-degree female relatives of someone with CD (such as a sister, mother, or daughter) are 14 times more likely to develop similar symptoms compared to women in the general population [7]. A shared environmental factor is believed to play a role in the development of the disorder. While conversion disorder can occur at any age, it most commonly appears during adolescence to early adulthood and is more prevalent in women [11].

Around two-thirds of patients with conversion disorder show signs of a psychiatric condition, with depression and trauma being the most frequently observed. Personality disorders are also commonly associated [12]. While there is no single known cause of CD, experts suggest that multiple risk factors and triggers may contribute to its onset. One of the more commonly reported triggers is the body's response to psychological trauma or a stressful event. Additionally, some doctors and researchers believe that physical injuries, infections, migraines, or panic attacks may also play a role in triggering the development of conversion disorder [2].

Many researchers are increasingly of the view that, regardless of the initial trigger, the symptoms of FNSD tend to become "stuck" rather than improving, leading to the development of functional problems [13]. Like many other disorders or diseases, FNSD has a variety of causes, risk factors, and a wide range of symptoms that can differ greatly from person to person. These symptoms can include vision loss, double vision, light sensitivity, limb weakness or paralysis, voice loss, slurred or stuttering speech, difficulty coordinating movements, memory issues, cognitive difficulties, headaches, migraines, loss of smell, chronic pain, numbness or tingling in the limbs, face, or body, hearing loss, seizures, fainting or blackouts, tremors, spasms, sleep disturbances, bladder issues, and even hallucinations [2].

Some patients experience only a few symptoms, while others may have many, with both the intensity and frequency of symptoms varying. In certain cases, symptoms are persistent, while in others, they can come and go. The exact number of people affected by FNSD is not well understood, though it is more common in developing countries [12]. In Western countries, the prevalence of conversion disorder (CD) has primarily been documented in neurology clinics, where it affects between 30% and 60% of patients. However, when considering the general population, estimates of the disorder's prevalence range from 0.011% to 0.5% [10].

In Pakistan, between 5% and 13% of all inpatient psychiatric admissions are linked to dissociative disorders, including conversion disorder. In patients at higher risk, maintaining a strong level of suspicion for this condition can lead to earlier identification, preventing unnecessary medical tests, avoiding invasive procedures, and conserving valuable healthcare resources [14]. In countries like Pakistan, negative attitudes toward mental health issues, coupled with sociocultural barriers, are key factors contributing to the lack of proper attention and the exacerbation of emotional and psychological disorders [15].

Our objective was to identify and analyse the various stressors contributing to the development of conversion disorder, as well as to examine the frequency and nature of both medical and non-medical consultations sought by affected individuals. In addition, we aimed to document the types and average number of symptoms reported by patients and to explore the prevailing local belief systems and cultural interpretations surrounding this illness. Understanding these factors is essential for improving diagnostic accuracy and guiding more effective, empathetic, and contextually appropriate management strategies. By delineating the psychosocial and cultural dimensions associated with conversion disorder, our research seeks to minimize unnecessary and costly investigations, reduce the burden on healthcare resources, and enhance patient outcomes. Moreover, the findings are intended to increase clinician awareness of how such disorders manifest within specific sociocultural settings, thereby informing more holistic and patient-centred approaches to care.

## Materials and methods

Data was collected from a cohort of 300 patients, in the span of two months, who either attended our outpatient psychiatry clinic directly or were referred by various departments, including the accident & emergency (A&E), rheumatology, pulmonology, neurology, cardiology, and other specialties. The patients were stratified into two primary groups, with 150 individuals in each, based on their mental health diagnoses, following the criteria set forth in the International Classification of Diseases (ICD-10). 

Group A comprised patients diagnosed with conversion disorder, while group B included individuals suffering from a range of other common psychiatric disorders. These groups were formed to allow a comparative analysis of the clinical, demographic, and therapeutic characteristics across different mental health conditions.

In conducting this study, we adhered rigorously to the ethical principles outlined in the Declaration of Helsinki 1975, as revised in 2008. All study procedures were carried out in accordance with the ethical standards set by both the relevant national regulatory bodies and the institutional committees overseeing human experimentation. The study protocol (IRB&EC/2024-GH/0133) was reviewed and approved by the Ethics Committee of the North West General Hospital and Research Centre, ensuring that all procedures involving human subjects met the required ethical guidelines.

Informed verbal consent was obtained from all participants prior to their inclusion in the study. This consent process ensured that each participant was fully aware of the nature of the study, their role in it, and their right to withdraw at any stage without any repercussions. It is worth noting that patients admitted to inpatient services were excluded from the study to maintain a focus on those attending outpatient care.

Data was systematically collected using pre-designed self-made questionnaires, which were structured to gather comprehensive demographic information, details on the symptoms experienced by the participants, their personal belief systems, and their history of prior interactions with healthcare providers. The questionnaires also sought to capture any previous engagement with physicians, psychiatrists, or a combination of both, as well as any use of alternative treatments such as consultations with spiritual healers. Statistical Product and Service Sciences (SPSS) version 22 (IBM Inc., Armonk, New York) was used to conduct the statistical analysis of the data.

## Results

**Figure 1:**
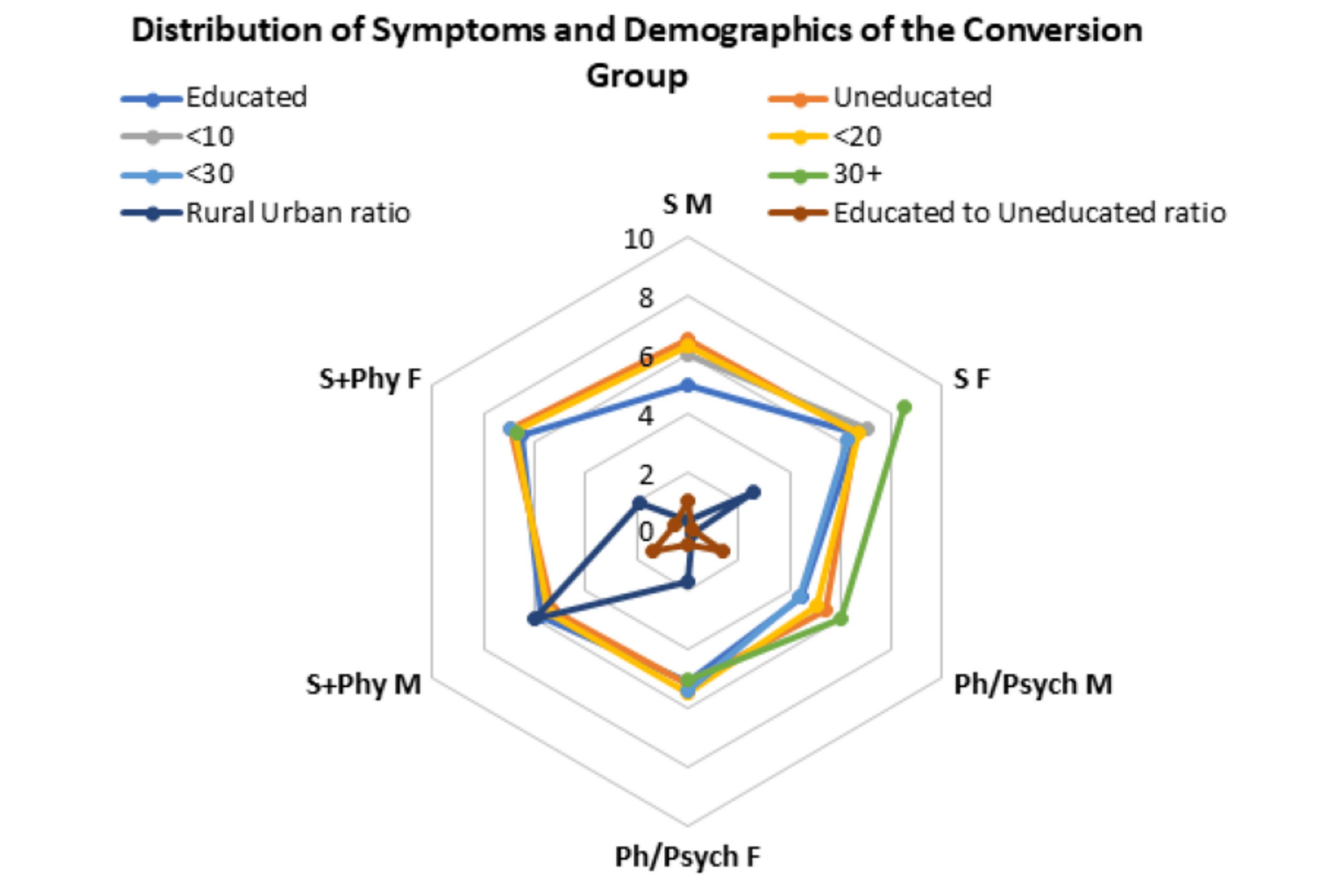
Group A demographics and gender-based distribution of average number of symptoms according to belief, education and age Ph/Psych Physical - psychiatric or physical cause for symptoms; S+Phy - spiritual and physical cause for symptoms; S - spiritual cause for symptoms; F - female; M - male; <10 - age group less than 10 years; <20 - age group between 10-20 years; <30 - age group between 20-30 years; 30+ - age group greater than 30 years.

Group A included 80% females (n=120) and 20% (n=30) males, with an average age of 24.3 years; 66.6% were uneducated, and 63.3% of them lived in rural areas (Figure 1). 69.33% (n=104) of the patients overall believed that there was a spiritual cause, with or without a physical/psychiatric cause, for their complaints. Females believed more in a spiritual cause with or without a physical/psychiatric cause for their symptoms, while most men believed in a physical cause alone (Pearson's Chi-squared value =16.1, p<0.001). 26.6% (n=40) believed in a spiritual cause alone for their complaints, with a female:male ratio (F:M) of 30:13.3%. 30.6% (n=46) believed their complaints to be wholly physical or psychiatric in nature, with a F:M of 22.5:63.3%. A combination of spiritual and physical/psychiatric causes for the presenting complaints was believed by 42.6% (n=64) of the group, with F:M of 47.5:23.3%.

In comparison, group B (n=150) included 54% females (n=81) and 46% males (n=69), with an average age of 36.5 years. 53.3% were educated and lived in urban areas.

Of group A, 81.3% experienced odd movements in the limbs as the most common symptom, followed by, clenching of the jaws (77.3%), dysphasia (76%), loss of consciousness (LOC; 73.3%), odd speech (69.3%), psychogenic nonepileptic seizure (PNES; 55.3%), odd behaviors (52.6%), headaches (50%), chest tightness (45.3%), other symptoms (19.3%), and weakness (18%)

In comparison, in group B, headaches were the most common symptom experienced in 66.6%, weakness (58.6%), other symptoms (45.3%), chest tightness (32.6%), and odd behaviors (21.3%). Behavioral outbursts (M:F of 27.5:16%) were more common in males compared to females.

In group A, patients who believed their complaints to be of a spiritual + physical/psychiatric nature had, on average, 6.77 symptoms, followed by the group believing in a spiritual/religious cause expressing 6.6 symptoms on average, and the physical/psychiatric group averaged 5.2 symptoms. Females presented with a higher number of symptoms over the three groups overall (Pearson's Chi-squared value of 21.8, p-value<0.0001). 68.3% had between six to eight symptoms, 25% had between three to five symptoms, while 6.1% presented with more than eight symptoms. The educated vs uneducated sample showed a higher number of symptoms in the uneducated group overall. with M:F of 5.6:6.4 in the educated group, and M:F of 4.9:6.2 in the uneducated group. Females from rural areas presented with a greater number of symptoms as compared to males (Pearson's Chi-squared value of 26.4, p-value<0.001), M:F of 5.4:6.6; whilst those from urban areas had a M:F of 5.1:6.1.

Historic trauma was identified in 70% of group A, recent arguments with family members as the most common trigger at 30.4%, followed by current relationship issues (20%), acute stress (19%), family tensions (17.1%), and marital problems (13.3%). In comparison, the patients in group B, 18% identified a previous trauma as a trigger, with acute stress as the most common trigger in 55.4%, followed by arguments with family (29.6%), and relationship issues (14.8%).

**Figure 2:**
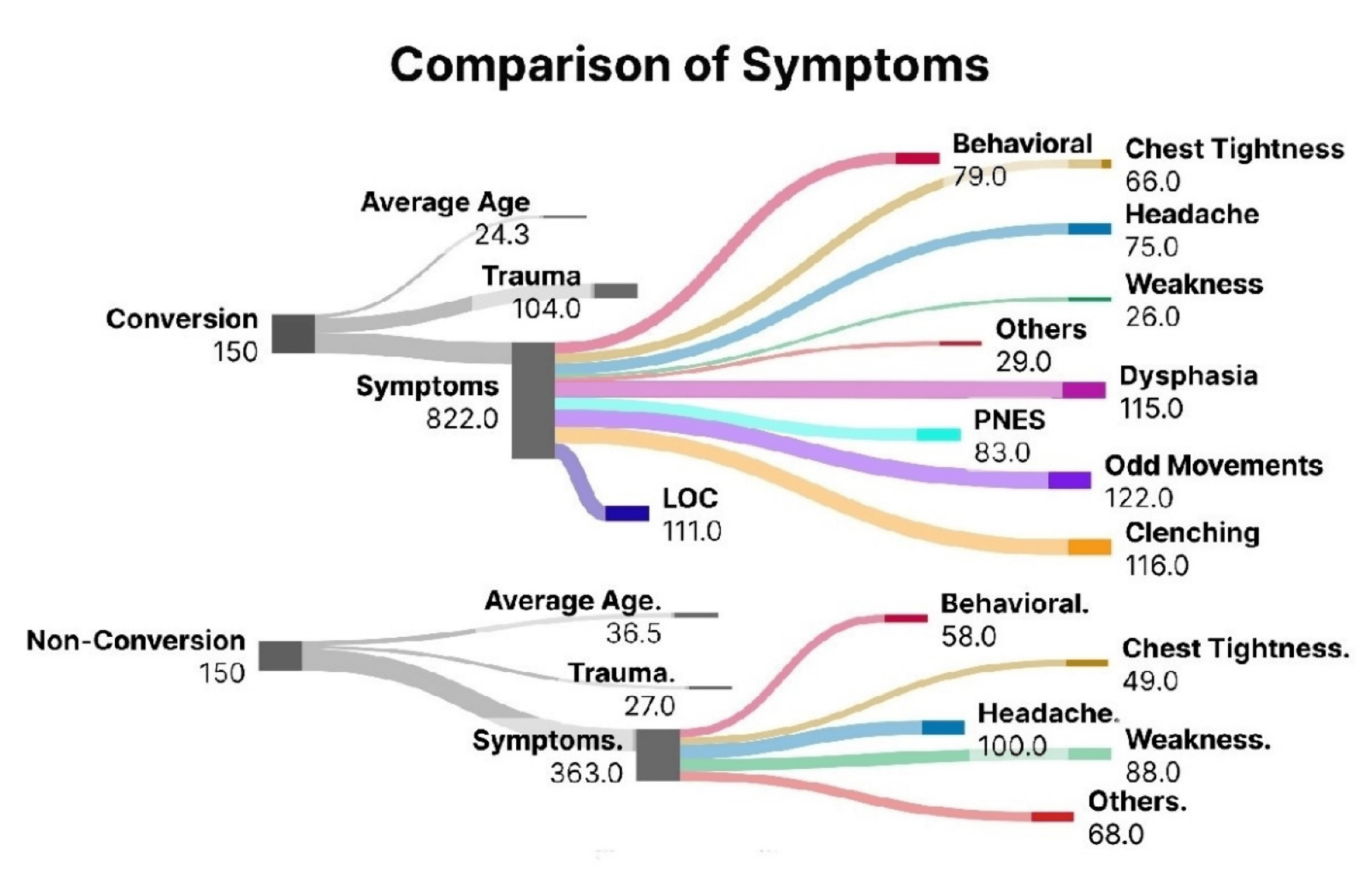
Comparative analysis of symptoms distribution between group A (conversion disorder) and group B (non-conversion). The total number of symptoms and each symptom are mentioned PNES - Psychogenic nonepileptic seizure; LOC - loss of consciousness

The highest number of individual symptoms on average was reported in group A, with marital problems at 6.7, followed by family-related tensions at 6.6, relationship issues at 6.28, acute stresses at 6.25, and arguments with family members at 6.03. In group B, relationship issues caused the highest number of average symptoms experienced at the rate of 3.25, followed by family arguments at 2.75, and acute stress at 2.46 symptoms. (Figure 2)

In males, age-wise distribution showed the highest number of symptoms (6.3 symptoms) in the 11-20 age group, who believed solely in a spiritual cause for their symptoms, followed by the less than 10 years age group with six symptoms on average. Patients over the age of 30 years presented with six symptoms on average, but believed them to be of a physical/psychiatric cause. The less than 20-year age group made up 63.3% of the group, and more than half (52.6%) of them believed their symptoms to be of a physical/ psychiatric cause only, with an average of 5.1 symptoms. 

Females aged more than 30 years showed the highest average number of symptoms at 8.5 in the group that believed their complaints to be of a solely spiritual cause, followed by the less than 10 years of age group with an average of seven symptoms. Those aged between 20-30 years who believed in a spiritual and physical/ psychiatric cause for their complaints averaged 6.94 symptoms, followed by the age group 10-20 years with 6.8 symptoms on average.

**Figure 3:**
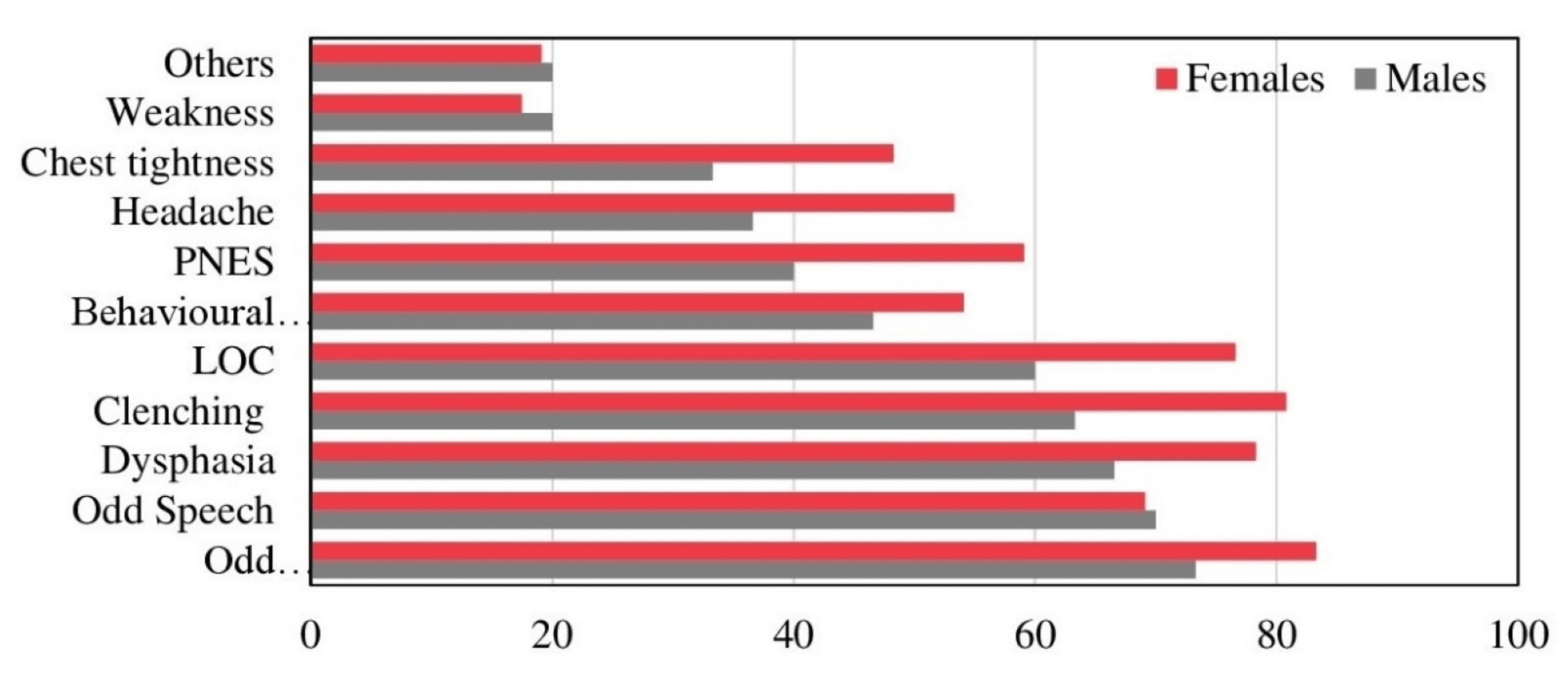
Percentage-wise distribution of symptoms in group A NES - Psychogenic nonepileptic seizure; LOC - loss of consciousness

In group A, 73.3% of males reported odd movements, as the most commonly experienced symptom followed by odd speech (70%), dysphasia (66.6%), jaw clenching (63.3%), LOC (60%), odd behaviors (46.6%), PNES (40%), headaches (36.6%), chest tightness (33.3%), weakness & other symptoms (20%). Most common symptom reported by females included odd movements (83.3%), clenching (80.8%), dysphasia (78.3%), LOC (76.6%), odd speech (69.1.%), PNES (59.1%), odd behaviors (54.1%), headaches (53.3%), chest tightness (48.3%), others (19.1%), and weakness (17.5%). Odd speech was reported almost equally in both sexes, while females experienced more of the symptoms overall, except for other symptoms and weakness, which were reported more in males (Figure 3).

In group B, Mixed anxiety and depression was the most common diagnosis with 42% sufferers, followed by Moderate depression (16%), panic attacks (6.6%), genialized anxiety disorder (6%), bipolar affective disorder (BPAD; 5.3%), Obsessive compulsive disorder (OCD; 4.6%), acute stress reaction, dementia, drug addiction and schizophrenia (2%), oppositional defiant disorder, acute psychotic episode and social anxiety (1.3%), pathological jealousy, post-natal depression, drug induced psychosis, learning disability, delirium, agoraphobia, adjustment disorder and abnormal grief reaction (0.6%).

In group B, 87.3% had made between one to three previous contacts with services, out of which 74.6% were psychiatric services, 20% with general physicians, 18.6% with both physicians and psychiatrists, 2% with spiritual/religious figures, and 2% with both a religious figure and a physician/psychiatrist. In comparison, group A showed 97.3% previous contact with services, out of which 25% had had at least four previous contacts, 44.6% of which were with other physicians, 32% with religious figures, 23.3% with psychiatrists, and 58% had had contact with both a religious figure and a physician or psychiatrist. Group A and group B were significantly associated with one to three and four to eight contacts, respectively (Pearson's Chi-squared value of 65, p<0.0001). Furthermore, group A and B showed a significant relationship with 0 and one to three psychiatric contacts, respectively (Pearson's Chi-squared value of 56.7, p<0.0001). Regarding both medical and religious services contact, group A was strongly associated with one to three contacts, and group B with 0 contacts (Pearson's Chi-squared value of 74.5, p<0.0001, and Pearson's Chi-squared value of 143.6, p<0.0001, respectively).

**Figure 4:**
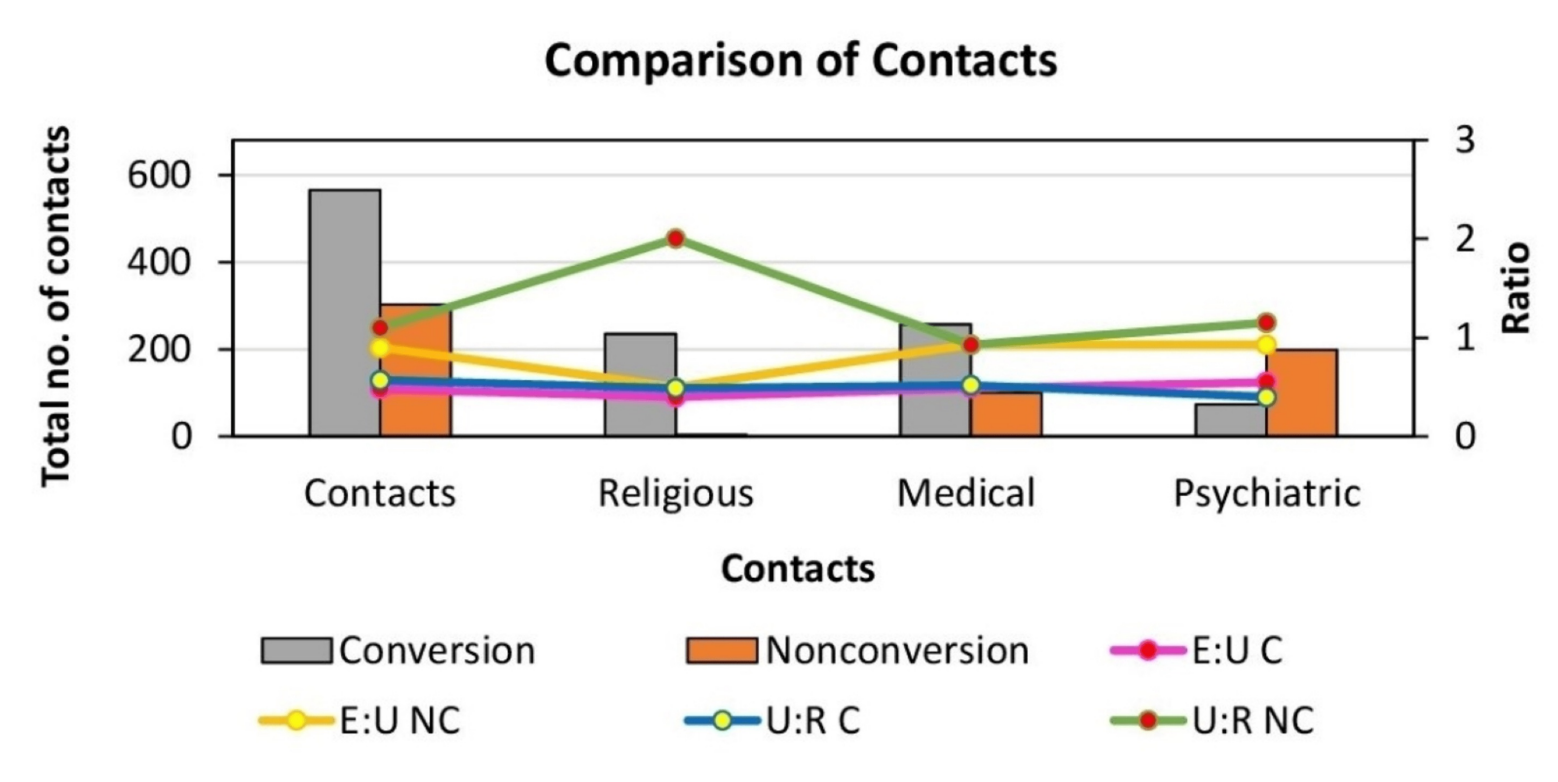
Comparative analysis of contact history between group A (conversion disorder) and group B (non-conversion) E:U - educated to uneducated ratio; U:R - urban to rural ratio; NC - non-conversion group; C - conversion group

Females presented with overall more symptoms on average, with a F:M of 2.5:2.2. A greater number of symptoms was noted in the uneducated group compared with the educated group (Pearson's Chi-squared value of 16.6, p<0.001), with M:F of 1.95:2.15 in the educated group, and M:F of 2.5:2.4 in the uneducated group. Females aged between 10-20 years who were uneducated presented with the highest number of symptoms at 3.2, (Pearson's Chi-squared value of 13.9, p<0.01), followed by the above 30 years educated group with three symptoms on average, the uneducated group above 30 years at 2.9, 11-20 years educated at 2.8, 20-30 years uneducated group at 2.4, 2-30 years educated with 2.3 and less than 10years educated with two symptoms on average (Figure 4).

Males uneducated, aged between 20-30, those above 30 years uneducated, and the educated group between 10-20 years presented with 2.5 symptoms on average. Less than 10 years educated and between 10-20 years uneducated both presented with two symptoms on average. Between 20-30 years educated group presented with 1.9 symptoms, and those above 30 years and educated had 1.6 symptoms on average. Males who resided in rural areas presented with more symptoms on average, compared to those living in urban settings (2.4:1.9), whilst females living in urban areas presented with more symptoms compared to their rural counterparts (2.8:2.3).

## Discussion

In Pakistani society, the cultural acceptance of physical symptoms and the high likelihood of seeking medical consultation have increased the tendency for emotional distress to manifest as dissociation and conversion disorder. This may help explain the higher incidence of conversion disorder in Asian countries, particularly Pakistan, in recent years [10]. In a country where basic needs are not always met, the provision of mental health services remains a distant goal.

Research on conversion disorder in both Western and non-Western countries has consistently found that it is more prevalent in cultures where conversion symptoms are seen as acceptable rather than unusual [16]. The incidence is notably higher in societies with strict, conservative social structures that discourage individuals from openly expressing their emotions [17]. Our study revealed that nearly 70% of those in the FNSD group attributed their symptoms to a culturally accepted explanation, often citing spiritual influences.

Western studies on the symptom patterns of conversion disorder have found that somatic complaints, such as headaches and lower back pain, are the most commonly reported symptoms [18]. Our study aligned with these findings, as headaches were reported by half of the participants. However, we also observed that abnormal limb posturing and jaw clenching were the most prevalent symptoms among patients with conversion disorder.

Around one-third to one-half of patients in psychiatric units here present with motor symptoms [19]. A study [12] also found that unresponsiveness and tremors (jerky movements) are among the most common symptoms of CD, which were both notably present in our population as well. In Pakistani culture, conversion disorder is often comorbid with anxiety and depressive symptoms [20]. In South Asian countries like Pakistan, India, and Bangladesh, the prevalence can be as high as 31%, with women being twice as likely to be affected as men [21,22], a trend confirmed in our study as well.

FNSD is reported to be more prevalent in rural populations, particularly among individuals with lower socioeconomic status, limited education, and reduced psychological awareness [6]. The higher incidence of conversion disorder in patients with a history of sexual or physical abuse is well-documented [23], and women are more frequently diagnosed with CD [4]. In Asian cultures, adherence to religious and cultural norms is considered virtuous and acceptable, and often serves as a way to express repressed emotions and desires [21].

Studies have shown that the prevalence of FNSD is higher among individuals who exhibit greater religiosity, lower literacy levels, and diminished socioeconomic status [24], and was confirmed by our study also. Developmental psychologists suggest that cultural socialization is passed down to the next generation, with certain behaviors being internalized and later displayed by children. This process may contribute to the development of FNSD in adulthood [25].

South Asia, with its strong ties to religious and cultural customs and a lower level of human development among the majority, is a prime example of this phenomenon [26].

Many medical professionals encounter patients whose symptoms are difficult to explain, often leading to feelings of hopelessness and frustration. As a result, there is a tendency to attribute unexplained physical symptoms to mental health issues. In medicine, terms like "supratentorial", "psychosomatic", and "functional" are often used to describe symptoms that lack a clear organic basis. Unfortunately, these terms are sometimes employed without fully considering their implications. In psychiatry, such symptoms are classified under somatoform disorders, which encompass a wide range of diagnoses, including conversion disorder [27].

One of the key limitations of our study is its reliance on data exclusively from the psychiatric services at a tertiary care hospital. While this setting provides valuable insights into the more complex cases of conversion disorder, it may not fully represent the broader spectrum of patients who may present with such disorders in secondary and primary care settings. Including data from these additional healthcare tiers would have offered a more holistic view of the prevalence and patterns of conversion disorder in the general population, especially among those who may not have access to specialized care.

Furthermore, the study would have been strengthened by a larger and more diverse sample size. Expanding the participant pool to include a wider range of demographics and geographical locations could have helped ensure greater generalizability of the findings. In addition, gathering data from multiple sources, such as general practitioners, community-based health workers, and other mental health professionals, would have provided a more comprehensive understanding of the disorder. This approach would also allow for the exploration of how conversion disorder is recognized and managed in various healthcare settings, further enhancing the robustness of our conclusions.

## Conclusions

Based on our study and despite the limitations of our study, we were able to gather all of the information that we had set out to, with good participation from participants in both groups. We were also able to replicate some of the data that had been collected from other researchers and reaffirm the data collected on the topic. Further robust research on this topic would help generate more information and possibly help in developing guidelines and protocols for treatment.

The identified presentations, the accompanying physical symptoms, and the underlying beliefs that underpin these complaints are all matters that we as medical and mental health providers need to be aware of. If we have a better grasp of the underlying ideas and cultural influences, we might be able to provide services that are better tailored to our patients' needs. Reducing needless investigations and referrals can help patients and their families stay focused on the psychological cause of their presentation. Examining the psychological causes of these symptoms is crucial, as is using a treatment strategy that incorporates physical therapy, psychology, and psychiatry.

Through public education, enlisting the assistance of spiritual healers, religious scholars, other primary care medical professionals, and doctors, we can do our part in educating the masses and reduce the impact such illnesses have on the health of the population as a whole and the burden on services.

## Appendices

**Table 1:** Comparison between Group A and B

Group A	M:F	Average Age	Education	Residence	Belief system	Symptoms	Trauma History	Psychiatric illness	Contacts
	120: 30	24.3yrs	66.6 %	63.3% rural	69.33% spiritual beliefs; 26.6% Spiritual cause alone, 30.6% physical/psychiatric cause alone, 42.6%combination of both spiritual and physical/psychiatric cause	Odd movements- 81.3%, jaw clenching- 77.3%, Dysphasia-76%, loss of consciousness or LOC 73.3%, odd speech 69.3%, Psychogenic nonepileptic seizure or PNES 55.3%, odd behaviors 52.6%, headaches 50%, chest tightness45.3%, other symptoms 19.3% and weakness 18%.	70%	100% Conversion disorder	97.3% previous contact with services; 25% at least 4 previous contacts (44.6% with other physicians, 32% with religious figures, 23.3% with psychiatrists and 58% had had contact with both a religious figure and a physician or psychiatrist)
Group B	M:F	Average Age	Education	Residence		Symptoms	Trauma History	Diagnosis	Contacts
	81:69	36.5yrs	53.3%	53.3% urban		Headaches 66.6%, weakness (58.6%), other symptoms (45.3%), chest tightness (32.6%) and odd behaviors (21.3%).	18%	Mixed anxiety and depression 42%, Moderate depression (16%), panic attacks (6.6%), genialized anxiety disorder (6%), Bipolar affective disorder or BPAD (5.3%), Obsessive compulsive disorder or OCD (4.6%), acute stress reaction, dementia, drug addiction and schizophrenia (2%), oppositional defiant disorder, acute psychotic episode and social anxiety (1.3%), pathological jealousy, post-natal depression, drug induced psychosis, learning disability, delirium, agoraphobia, adjustment disorder and abnormal grief reaction (0.6%).	87.3% had between 1-3 previous contacts with services (74.6% were psychiatric services, 20% with general physicians, 18.6% with both physicians and psychiatrists, 2% with spiritual/religious figures, and 2% with both a religious figure and a physician/psychiatrist).
